# Accumulation of DNA damage alters microRNA gene transcription in *Arabidopsis thaliana*

**DOI:** 10.1186/s12870-022-03951-9

**Published:** 2022-12-12

**Authors:** Juan Du, Yang Liu, Lu Lu, Jianfei Shi, Longqian Xu, Qi Li, Xiaofei Cheng, Jinfeng Chen, Xiaoming Zhang

**Affiliations:** 1 State Key Laboratory of Integrated Management of Pest Insects and Rodents, Institute of Zoology, Chinese Academy of Sciences, Beijing, 100101 China; 2 CAS Center for Excellence in Biotic Interactions, University of Chinese Academy of Sciences, Beijing, 100049 China; 3 Department of Life Sciences, Henan Normal University, Xinxiang, Henan 453007 China; 4 Key Laboratory of Germplasm Enhancement, Physiology and Ecology of Food Crops in Cold Region of Chinese Education Ministry, College of Agriculture, Northeast Agricultural University, Harbin, Heilongjiang, 150030 China

**Keywords:** DNA damage repair, miRNA, ZDP, APE2, FPG

## Abstract

**Background:**

MicroRNAs (miRNAs) and other epigenetic modifications play fundamental roles in all eukaryotic biological processes. DNA damage repair is a key process for maintaining the genomic integrity of different organisms exposed to diverse stresses. However, the reaction of miRNAs in the DNA damage repair process is unclear.

**Results:**

In this study, we found that the simultaneous mutation of *zinc finger DNA 3′-phosphoesterase* (*ZDP*) and *AP endonuclease 2* (*APE2*), two genes that play overlapping roles in active DNA demethylation and base excision repair (BER), led to genome-wide alteration of miRNAs. The transcripts of newly transcribed miRNA-encoding genes (*MIR*s) decreased significantly in *zdp*/*ape2,* indicating that the mutation of *ZDP* and *APE2* affected the accumulation of miRNAs at the transcriptional level. In addition, the introduction of base damage with the DNA-alkylating reagent methyl methanesulfonate (MMS) accelerated the reduction of miRNAs in *zdp*/*ape2*. Further mutation of *FORMAMIDOPYRIMIDINE DNA GLYCOSYLASE* (*FPG*), a bifunctional DNA glycosylase/lyase, rescued the accumulation of miRNAs in *zdp/ape2*, suggesting that the accumulation of DNA damage repair intermediates induced the transcriptional repression of miRNAs.

**Conclusions:**

Our investigation indicates that the accumulation of DNA damage repair intermediates inhibit miRNAs accumulation by inhibiting *MIR* transcriptions.

**Supplementary Information:**

The online version contains supplementary material available at 10.1186/s12870-022-03951-9.

## Background

The cellular DNA of living species is normally damaged by endogenous and exogenous genotoxins. DNA bases are particularly susceptible to oxidation mediated by reactive oxygen species (ROS) [1]. The most thoroughly examined oxidized base product is 7,8-dihydro-8-oxoguanine (8-oxoG), which is produced due to the lower redox potential of guanine [1, 2]. Failure to remove 8-oxoG results in G-to-T mutations [1]. Due to their sessile and photoautotrophic properties, plants are vulnerable to be attacked by ROS derived from photosynthesis and defence responses to biotic and abiotic stress [3, 4]. Base excision repair (BER) is essential for repairing a wide range of lesions, including alkylation, deamination, oxidation and apurinic/apyrimidinic (AP) site lesions resulting from spontaneous depurination or processing of blocked 3′-ends of single-strand breaks, and BER is involved in active DNA demethylation to maintain balanced DNA methylation patterns [5–7]. The BER pathway in *Arabidopsis* is initiated by DNA glycosylases, which remove modified bases by cleaving the N-glycosidic bond and generate an abasic site without disruption to the phosphate-sugar DNA backbone [8, 9]. FORMAMIDOPYRIMIDINE DNA GLYCOSYLASE (FPG) and 8-oxoguanine DNA GLYCOSYLASE 1 enzymes show glycosylase/lyase activities that initiate the repair of oxidized 8-oxoG in plants [10, 11]. Subsequently, the abasic sites are processed by enzymes with AP lyase activity or by AP endonucleases, producing either unconventional 3′-phospho-α,β-unsaturated aldehyde (3′-PUA) or 3′-phosphate (3′-P) and 5′-hydroxyl (OH) termini, or by cleaving the 5′ DNA backbone to generate 3′-OH and 5′-deoxyribose-5-phosphate (5′-dRP) ends [12–15]. Unconventional 3′-PUA or 3′-P and 5′-dRP ends need to be converted to conventional 3′-OH and 5′-P termini to enable subsequent polymerization and ligation. However, the molecular mechanism of BER in plants is unclear.

Zinc finger DNA 3′-phosphoesterase (ZDP) removes 3′-P group to provide 3′-OH end [10, 16]. *Arabidopsis thaliana* encodes three AP endonucleases proteins, APE1L, APE2 and ARP [17]. APE1L and ARP play vital roles in removing the 3′-PUA group, and APE2 shows the weakest AP endonuclease activity [17]. Active DNA demethylation in plants removes methylated cytosine through the BER pathway [18]. The excision of methylated cytosine by REPRESSOR of SILENCING 1 (ROS1)/DEMETER generates gapped DNA intermediates with blocked 3′-end (3′-PUA or 3′-P) [12, 19, 20]. Genetic and biochemical analyses have indicated that both ZDP and APE1L interact with ROS1 and function downstream of ROS1 in active DNA demethylation pathway [18, 19]. Simultaneous mutation of *APE1L* and *ZDP* leads to DNA hypermethylation in multiple genes and embryonic lethality [18]. APE2 shows 3′-phosphatase activity that overlaps with that of ZDP for converting 3′-P end to 3′-OH end during BER and active DNA demethylation [16, 21–23]. The simultaneous mutation in *ZDP* and *APE2* causes the accumulation of unrepaired 3′-blocked DNA. Comet assays showed a great increase in the DNA damage signal in *zdp-1*/*ape2-2* compared with that in Col-0 under normal conditions [16]. Unrepaired DNA lesions and intermediates initiate the DNA damage response (DDR), which transcriptionally regulates multiple genes controlling cell cycle checkpoints, DNA repair and programmed cell death [24–26]. In animals and plants, double-strand breaks and single-strand breaks are sensed by the MRE11-RAD50-NBS1 complex and RPA/Rad9-Hus1-Rad1 complex, respectively, each of which recruits the kinase proteins ataxia telangiectasia mutated (ATM) and ATM-related and Rad3-related to trigger DDR [27–31]. The accumulation of unrepaired 3′-blocked DNA leads to cytological differences, including different cell sizes, cell numbers and root meristem structures [26].

Plant miRNAs are 21–24 nucleotide (nt) small RNAs (sRNAs) that control development, immunity, metabolism, and other biological processes [32]. The transcription of miRNA-encoding genes (*MIR*s) and processing of primary miRNAs (pri-miRNAs) are coupled in the nucleus [33–35]. Pri-miRNAs are processed into stem-loop precursors and then into miRNA/miRNA* duplexes via the Dicing complex [36, 37]. Mature miRNA duplexes are mainly loaded into ARGONAUTE 1 (AGO1), which induces the transcriptional or posttranscriptional repression of target genes [38]. miRNA responses to DNA damage and the regulatory roles played by miRNAs in DNA damage repair and the DDR have been frequently reported in mammals [39]. For instance, miR-421 and miR-100 has been reported to suppress *ATM* expression by targeting the 3′ UTR of *ATM* transcripts [40, 41]. The ATM kinase induced miRNA biogenesis by increasing pri-miRNA processing in mouse embryonic fibroblasts [42]. Moreover, deletion of Dicer in the developing mouse cerebellum resulted in accumulation of DNA damage [43]. In addition, the transcription factors E2F and Myc induced the transcription of miR-17–92, which was then post-transcriptionally inhibited by miR-17–92 in return, forming a feedback loop in a cancer network [44]. However, the roles of miRNAs in plant DNA damage repair and the DDR are unknown. The sRNA and degradome sequencing data analysis revealed that *XPB2*, a DNA repair helicase, was targeted by tae-miR1122c-3p in male sterile wheat lines [45]. *MRE11*, the gene encoding a DNA repair and meiosis protein, was putatively targeted by miR5261 in *Citrus sinensis* [46]. Based on recent studies, an interrelation between redox balance, the DDR, and miRNAs has been proposed [47]. However, studies on miRNA responses to DNA damage are scarce and preliminarily data have been primarily obtained via sRNA sequencing analysis. Through deep-sequencing profiling, 58 miRNAs responding to DNA damage and 41 corresponding potential target genes related to DNA repair have been predicted [48]. In summary, the miRNA response to DNA damage remains elusive in plants.

The simultaneous mutation of *ZDP* and *APE2* results in severe developmental phenotypes, including retarded root growth and slightly serrated leaves [16], which implies that miRNAs may be differentially expressed in *zdp*/*ape2*. In this study, we studied the reaction of plant miRNAs in response to DNA damage. We identified a genome-wide alteration of the miRNA population in *zdp-1*/*ape2-2* mutant plants. The decrease in miRNAs was caused by reduced *MIR* transcription in *zdp*/*ape2* mutant plants. We then found that the accumulation of DNA damage repair intermediates induced the transcriptional repression of *MIRs*. Our observations thus reveal that plant miRNAs react to DNA damage.

## Results

### Simultaneous mutation of *ZDP* and *APE2* leads to genome-wide alteration of miRNAs

**Fig. 1:**
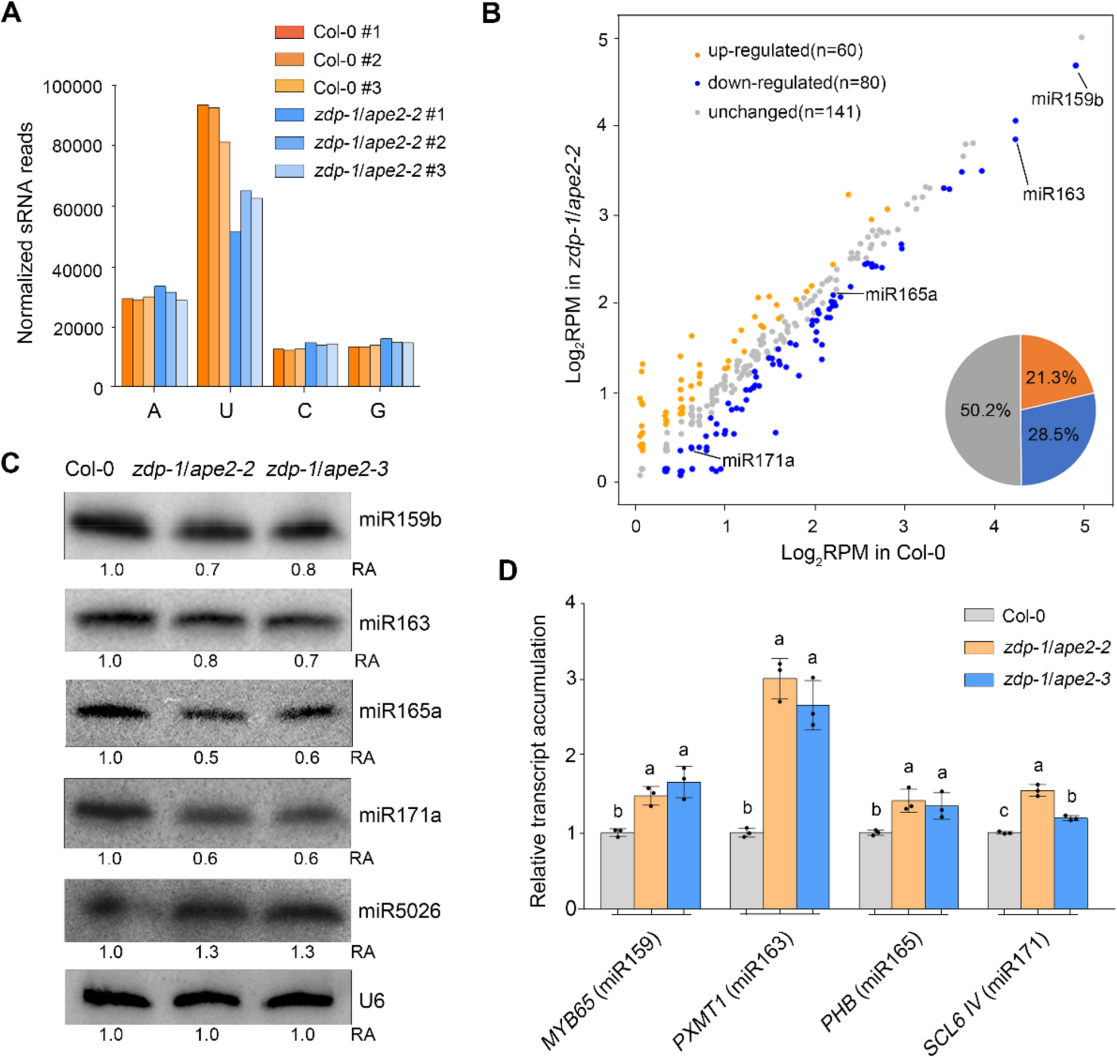
The simultaneous mutation of *ZDP* and *APE2* leads to genome-wide alteration of miRNAs. **A** The relative frequency analysis of the 5′-terminal nucleotide of miRNAs in Col-0 plants and *zdp-1*/*ape2-2* mutant plants. **B** Mean abundance analysis in *zdp-1*/*ape2-2* mutant compared with Col-0 plants. The blue, yellow and pale colours represent downregulated, upregulated and unchanged miRNAs in *zdp-1*/*ape2-2* mutant compared to Col-0 plants, respectively. **C** Northern blots showing the accumulation of miRNAs. U6 was the RNA loading control. RA indicates the relative fold change of miRNAs in *zdp-1*/*ape2-2* and *zdp-1*/*ape2-3* mutant compared with those in Col-0 plants. **D** Relative transcript levels of miRNA targets in Col-0, *zdp-1*/*ape2-2*, and *zdp-1*/*ape2-3* plants. *EF1α* was used as the internal control. Statistically significant differences between different genotypes are indicated by different lower-case letters (*P* < 0.05, one-way ANOVA per gene, performed separately)

To assess whether the developmental defects in *zdp*/*ape2* are accompanied with the differentially expressed miRNAs, we first determined the accumulation alterations of sRNAs by sRNA sequencing. sRNA libraries based on 3 biological repeats were established with seedlings from 2-week-old Col-0 and *zdp-1*/*ape2-2* plants. After adapter trimming and low-quality read filtering, 29,553,625, 27,290,680, 27,636,402, 27,945,355, 30,965,677, and 30,281,874 clean sRNA reads were obtained from the Col-0 and *zdp-1*/*ape2-2* libraries, respectively (Supplementary Fig. S1a, Supplementary Table S1). Clean sRNA reads were mapped against the TAIR10 *Arabidopsis* genome, and only unique mapping reads with perfect match were retained for further analyses. The lengths of sRNAs peaked at 21- and 24-nt in both the Col-0 and *zdp-1*/*ape2-2* plants. However, the abundance of 21-nt and 24-nt sRNAs was reduced by ~ 16.7% and ~ 15.9% in the *zdp-1*/*ape2-2* mutant (Supplementary Fig. S1b, Supplementary Table S2). Analysis of 5′-terminal nucleotide preferences revealed that the abundance of 21-nt sRNAs with uracil (U) at the 5′-terminus was significantly decreased to ~ 66.9% in *zdp-1*/*ape2-2* mutant (Fig. 1a). Because most plant miRNAs are 21-nt sRNAs with a 5′-terminal uridine and associate with AGO1 [38, 49], these results suggest that the accumulation of miRNAs may have been lower in *zdp-1*/*ape2-2* than that in Col-0. We quantified the miRNA abundance from sRNA reads and found that the accumulation of 80 (28.5%) miRNAs decreased in *zdp-1*/*ape2-2* mutant compared to Col-0 plants (Fig. 1b, Supplementary Fig. S1c, Supplementary Table S3). The levels of miRNAs related to development (e.g., miR159b, miR163, miR165a, and miR171a) were significantly reduced in the *zdp-1*/*ape2-2* mutant (Fig. 1b, Supplementary Table S3) [50]. Northern blot analysis revealed that the accumulation of miR159b, miR163, miR165a, and miR171a, but not miR5026 that associated with AGO2, decreased in *zdp-1*/*ape2-2* and *zdp-1*/*ape2-3* mutant plants (Fig. 1c, Supplementary Fig. S2). Moreover, the transcript levels of *MYB65* (a target of miR159) [51], *PXMT1* (a target of miR163) [52], *PHB* (a target of miR165/6) [53], and *SCL6 IV* (a target of miR171) [54] increased in the *zdp-1*/*ape2-2* and *zdp-1*/*ape2-3* mutant plants (Fig. 1d, Supplementary Table S5). AtAGO1 selectively binds miRNAs, and dysfunction in AtAGO1 leads to severe developmental defects [55, 56]. Therefore, we crossed *ago1-27* mutant with the *zdp-1*/*ape2-2* double mutant plants to create triple mutant plants. The triple homozygous mutants exhibited much more severe developmental defects, including smaller plant size and narrower leaves (Supplementary Fig. S3), which further indicates the roles for ZDP and APE2 in miRNA functions.

### Mutation of *ZDP* and *APE2* decreases *MIR* transcription

**Fig. 2:**
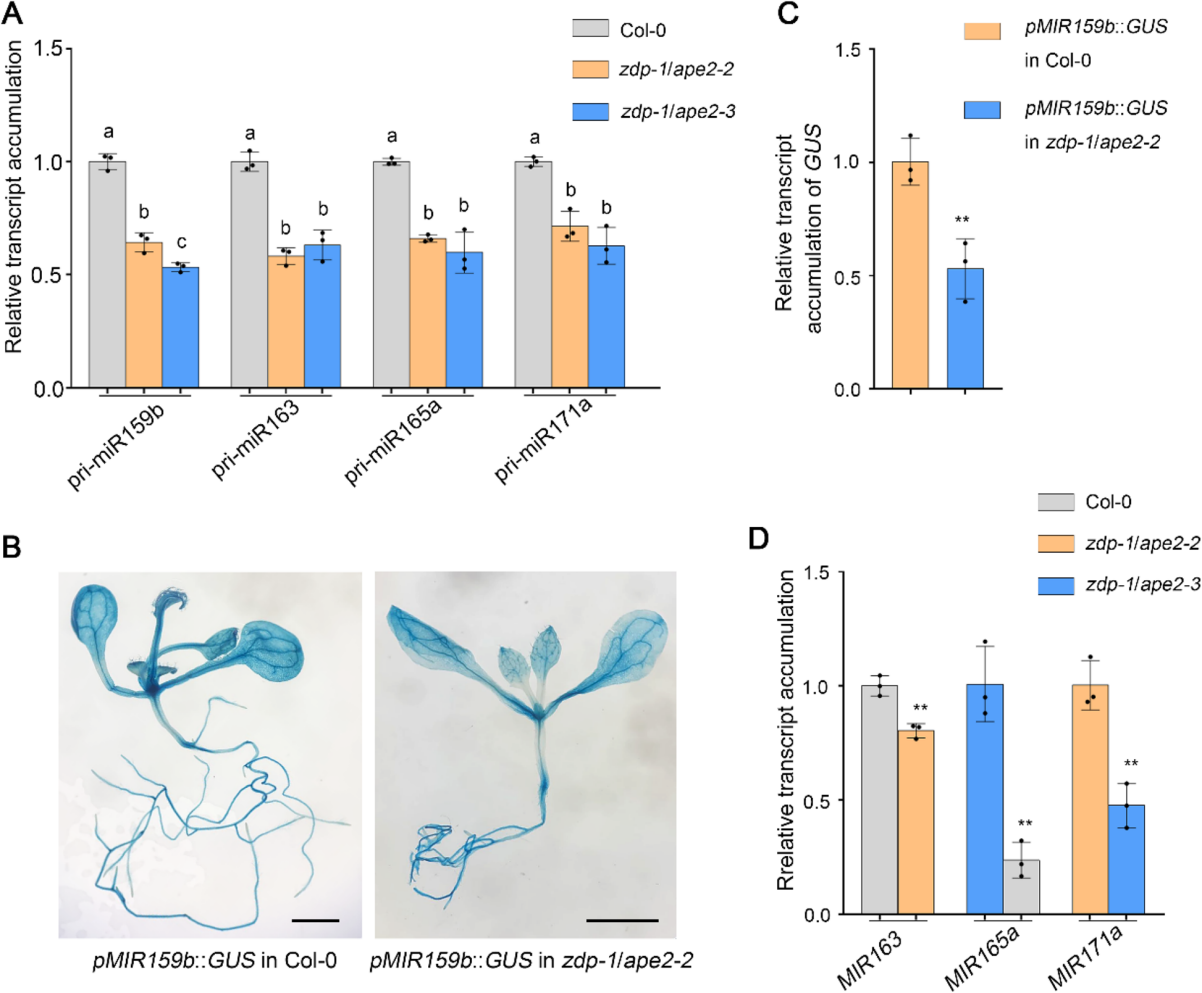
ZDP and APE2 promote the transcription of *MIR* genes. **A** Transcript levels of 4 pri-miRNAs in 2-week-old seedlings of Col-0, *zdp-1*/*ape2-2*, and *zdp-1*/*ape2-3* plants. *EF1α* was used as the internal control. Error bars represent standard deviation calculated on the basis of 3 independent replicates. Statistically significant differences between different genotypes are indicated by different lower case letters (*P* < 0.05, one-way ANOVA per gene, performed separately). **B** GUS staining of samples harbouring a *pMIR159b*::*GUS* in Col-0 or *zdp-1*/*ape2-2* background. Scale bar, 0.2 cm. **C** *GUS* transcript level was determined by RT‒qPCR in samples harbouring a *pMIR159b*::*GUS* in Col-0 and *zdp-1*/*ape2-2* background. *EF1α* was used as the internal control. Asterisks indicate significant differences in *GUS* expressions between plants with Col-0 and *zdp-1*/*ape2-2* mutant background (Student's *t* test, **P < 0.01). **D** Transcription rates of *MIR163*, *MIR165a*, and *MIR171a* in Col-0 and *zdp-1*/*ape2-2* plants, as measured by nuclear run-on assay and RT‒qPCR. *EF1α* was used as the internal control. Asterisks indicate significant differences in relative expression level of *MIR* transcripts between Col-0 and *zdp-1*/*ape2-2* plants (Student's *t* test, **P < 0.01)

Next, we set out to determine the molecular mechanism through which dysfunction of *ZDP* and *APE2* decreases miRNA accumulation. As miRNAs are processed from *MIR-*encoded pri-miRNAs by Dicing complex [36, 37], we compared the abundance of pri-miRNAs in Col-0, *zdp-1*/*ape2-2*, and *zdp-1*/*ape2-3* plants. The RT‒qPCR assay showed that the relative transcript levels of pri-miR159b, pri-miR163, pri-miR165a, and pri-miR171a in the *zdp-1*/*ape2-2* and *zdp-1*/*ape2-3* double mutant plants were ~ 20.8%—48.8% lower than those in Col-0 plants (Fig. 2a, Supplementary Table S6). These results indicate that *ZDP* and *APE2* promote pri-miRNA accumulation and thus enhance miRNA accumulation.

The decreased accumulation of pri-miRNAs and miRNAs in *zdp*/*ape2* double mutant plants may have been caused by inhibited transcription of *MIR*s. To assess this possibility, we crossed *zdp-1*/*ape2-2* mutant with a GUS reporter line under the control of the *MIR159b* promoter (*pMIR159b::GUS*) and obtained homozygous *pMIR159b::GUS in zdp-1*/*ape2-2* plants [57]. GUS staining revealed that GUS activity was lower in *pMIR159b::GUS in zdp-1*/*ape2-2* than that in *pMIR159b::GUS* in Col-0 plants (Fig. 2b). In addition, RT‒qPCR analysis showed that the relative expression of *GUS* transcripts decreased by ~ 53.1% in the *pMIR159b::GUS in zdp-1*/*ape2-2* compared with *pMIR159b::GUS* in Col-0 plants (Fig. 2c, Supplementary Table S6), which indicates that dysfunctional *ZDP* and *APE2* led to the decrease in the transcription of *MIR*s. To confirm the positive role of ZDP and APE2 on *MIR* transcription, newly transcribed RNA transcripts were detected by nuclear run-on assays. RT‒qPCR assays revealed that the abundance of newly transcribed *MIR163*, *MIR165a*, and *MIR171a* transcripts decreased significantly in the *zdp-1*/*ape2-2* mutant (Fig. 2d, Supplementary Table S6). These results suggest that dysfunction of *ZDP* and *APE2* decrease the transcription of *MIR*s, which leads to the reduced accumulation of pri-miRNAs and mature miRNAs.

### Dysregulated DNA damage repair reduces *MIR* transcription

We then continued to determine the underlying mechanism by which the dysfunction of ZDP and APE2 decreases *MIR* transcription. As ZDP and APE2 play dual roles in active DNA demethylation and DNA damage repair [16, 26], the decreased transcription of *MIR*s in *ZDP* and *APE2* mutant plants may be caused by the altered accumulation of DNA methylation or damaged DNA. To test these possibilities, we measured the methylation levels on *MIR*s by analysing bisulfite sequencing data obtained from Col-0 and *zdp-1*/*ape2-2* plants [16]. No significant difference in methylation level was found for *MIR159b*, *MIR163*, *MIR165a*, or *MIR171a* (Supplementary Fig. S4). Thus, the active DNA demethylation activity of ZDP and APE2 was likely not the cause of the transcriptional regulation of these *MIR*s.

**Fig. 3:**
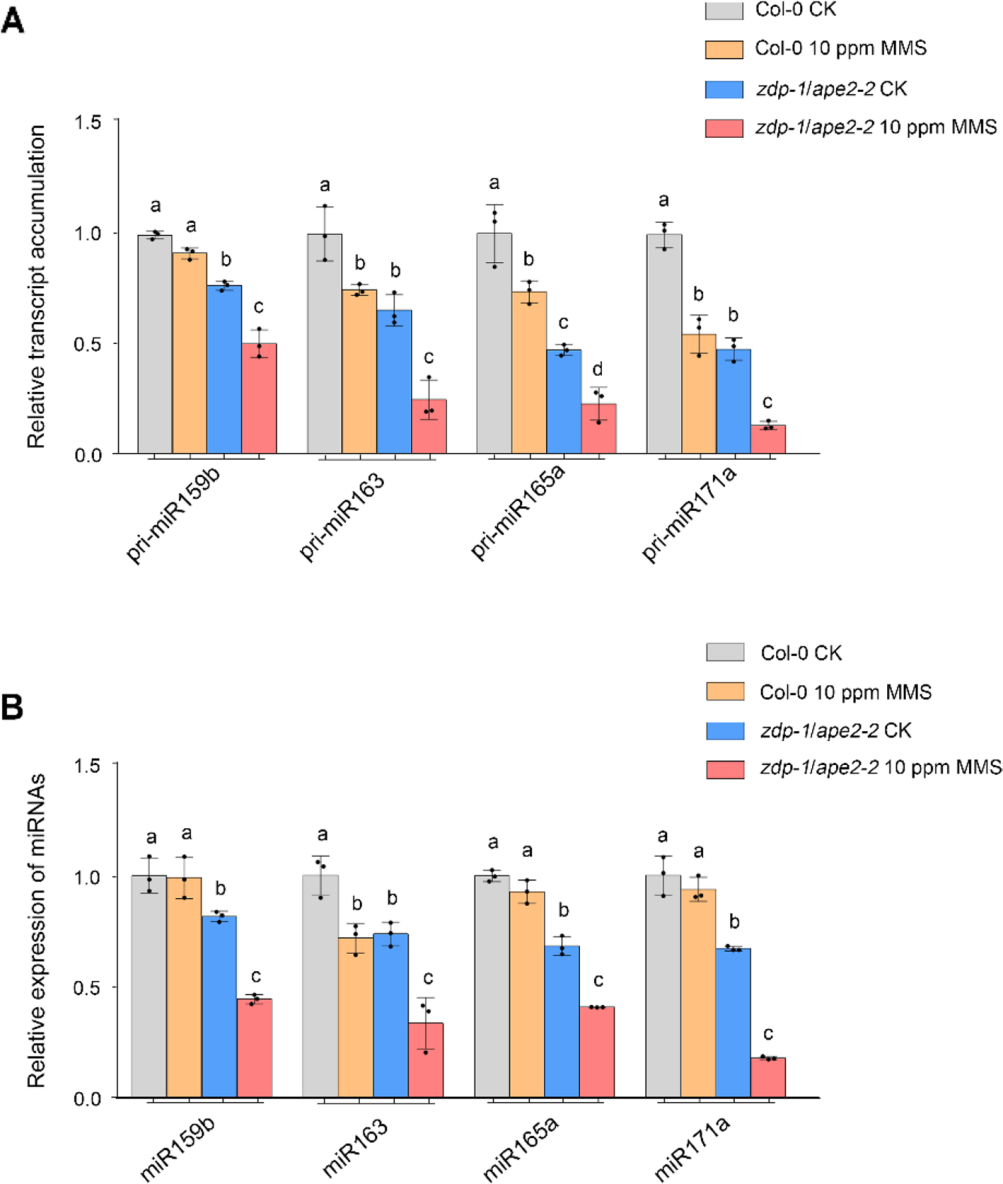
*MIR* transcription is inhibited by MMS treatment in *zdp*/*ape2* mutants. **A** RT‒qPCR detection of the relative expression levels of pri-miRNAs in Col-0 and *zdp-1*/*ape2-2* mutant plants treated with 0 ppm or 10 ppm MMS. EF1α was used as the internal control. Error bars represent the standard deviation calculated on the basis of 3 independent replicates. Statistically significant differences between different genotypes are indicated by different lower-case letters (*P* < 0.05, one-way ANOVA per gene, performed separately). **B** RT‒qPCR detection of the accumulation of miRNAs in Col-0 and *zdp-1*/*ape2-2* plants treated with 0 ppm or 10 ppm MMS. *U6* was used as the internal control. Error bars represent the standard deviation calculated from 3 independent replicates. Statistically significant differences between different genotypes are indicated by different lower-case letters (*P* < 0.05, one-way ANOVA per gene, performed separately)

As ZDP and APE2 play overlapping roles in BER [26], we wondered whether ZDP and APE2 affect *MIR* transcription through the DNA damage repair pathway. Small chemical alterations in DNA bases and single-strand DNA breaks are targeted by BER. We therefore treated Col-0 with the DNA alkylating reagent MMS to increase damaged bases. After 10 ppm MMS treatment, a mild reduction in pri-miR159b, pri-miR163, pri-miR165a, and pri-miR171a accumulation was observed in the Col-0 plants (Fig. 3a, Supplementary Table S7), indicating that DNA damage decreases *MIR* transcription. The *zdp-1*/*ape2-2* mutant was also treated with 10 ppm MMS. RT‒qPCR assays showed that the accumulation of pri-miRNAs in the MMS treated *zdp-1*/*ape2-2* was significantly lower than that in the control *zdp-1*/*ape2-2* mutant plants (Fig. 3a, Supplementary Table S7). Moreover, the accumulation of pri-miRNAs in MMS-treated *zdp-1*/*ape2-2* decreased dramatically compared with that in MMS-treated Col-0 (Fig. 3a, Supplementary Table S7). Moreover, the levels of mature miRNAs decreased significantly in the *zdp-1*/*ape2-2* mutant after 10 ppm MMS treatment (Fig. 3b, Supplementary Table S7). These results indicate that the reduction in miRNAs in the *zdp*/*ape2* mutant can be attributed to the malfunction of ZDP and APE2 in DNA damage repair pathway.

### DNA repair intermediate accumulation decreases *MIR* transcription

**Fig. 4:**
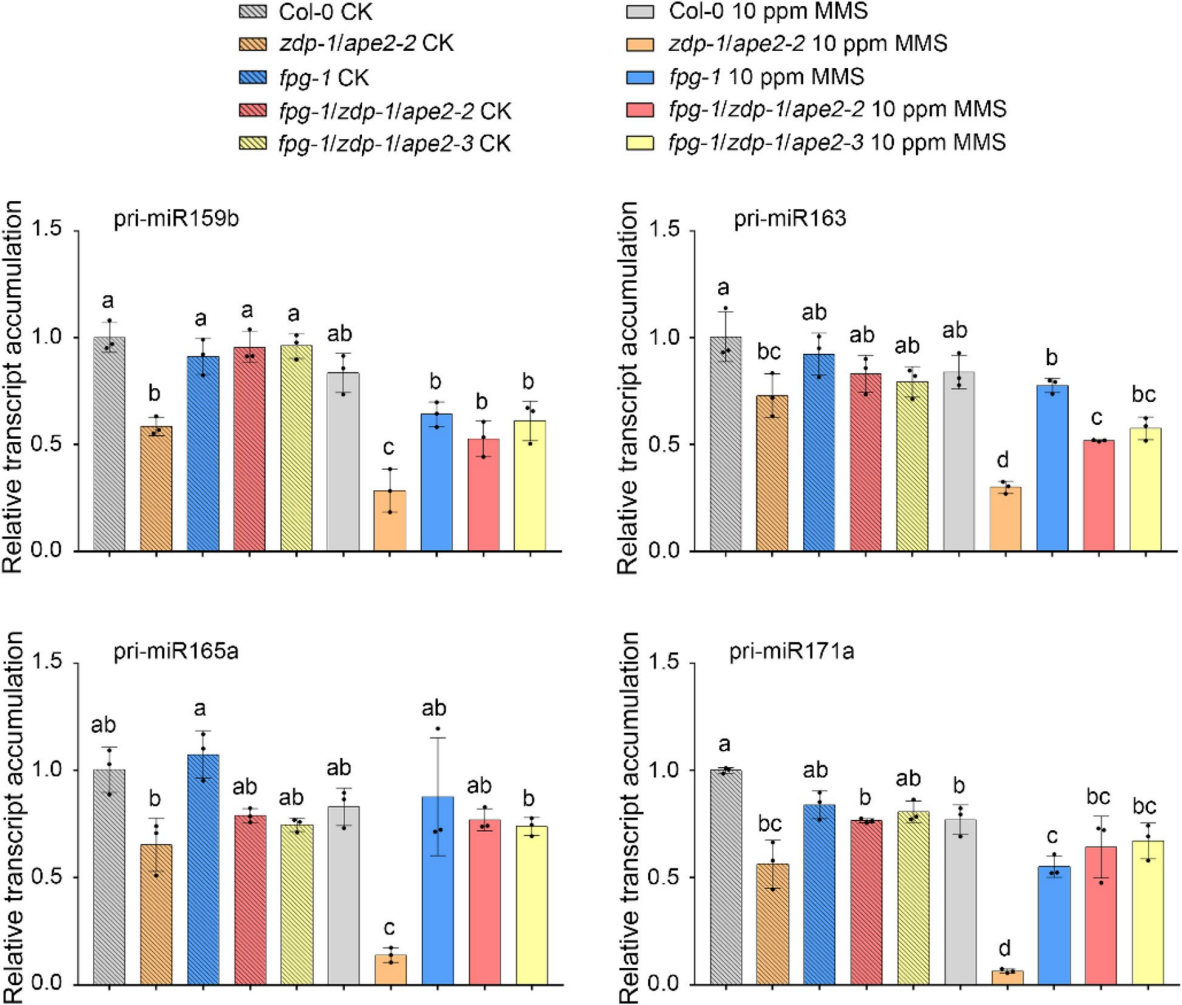
The mutation of *FPG* on *zdp*/*ape2* background rescues the accumulation of pri-miRNAs. RT‒qPCR detection of the relative expression levels of pri-miRNAs in Col-0, *zdp-1*/*ape2-2*, *fpg-1*, *fpg-1*/*zdp-1*/*ape2-2*, and *fpg-1*/*zdp-1*/*ape2-3* plants treated with 0 ppm or 10 ppm MMS. *EF1α* was used as the internal control. Error bars represent standard deviation calculated from 3 independent replicates. Statistically significant differences between different genotypes are indicated by different lower-case letters (*P* < 0.05, one-way ANOVA per gene, performed separately)

ZDP and APE2 play overlapping roles in BER by transforming the AP 3′-PUA or 3′-P end to form 3′-OH termini [16, 21–23, 26]. The increased DNA damage signal detected in the *zdp-1*/*ape2-2* mutant induces expression of genes involved in the DDR [16]. In plants, FPG is a bifunctional DNA glycosylase/lyase that produces 3′-P ends during BER and is critical for repairing 8-oxoG and AP sites created by MMS [10, 26]. To determine whether the accumulation of 3′-blocked DNA repair intermediates participate in *MIR* transcription regulation, we mutated *FPG* in *zdp*/*ape2* background to prevent the production of 3′-blocked DNA repair intermediates in *zdp*/*ape2* mutant. RT‒qPCR assays were performed to examine pri-miRNA accumulation in Col-0, *zdp-1*/*ape2-2*, *fpg-1*, *fpg-1*/*zdp-1*/*ape2-2*, and *fpg-1*/*zdp-1*/*ape2-3* plants treated with 0 or 10 ppm MMS. The relative expression level of the pri-miRNAs in the *fpg-1* mutant was similar to that in the Col-0 (Fig. 4, Supplementary Table S8). In addition, the accumulation of pri-miRNAs were comparable between *fpg-1*/*zdp-1*/*ape2-2*, *fpg-1*/*zdp-1/ape2-3* and *fpg-1* plants with or without MMS treatment (Fig. 4, Supplementary Table S8). A previous study suggested that the introduction of mutation in *FPG* strongly recovered the developmental defects in *zdp/ape2* mutant [26]. Taken together, these results indicate that FPG dysregulation prevents the accumulation of DNA repair intermediates in *zdp*/*ape2* mutant, and that the accumulation of 3′-blocked DNA repair intermediates decrease the plant miRNA transcription.

## Discussion

**Fig. 5:**
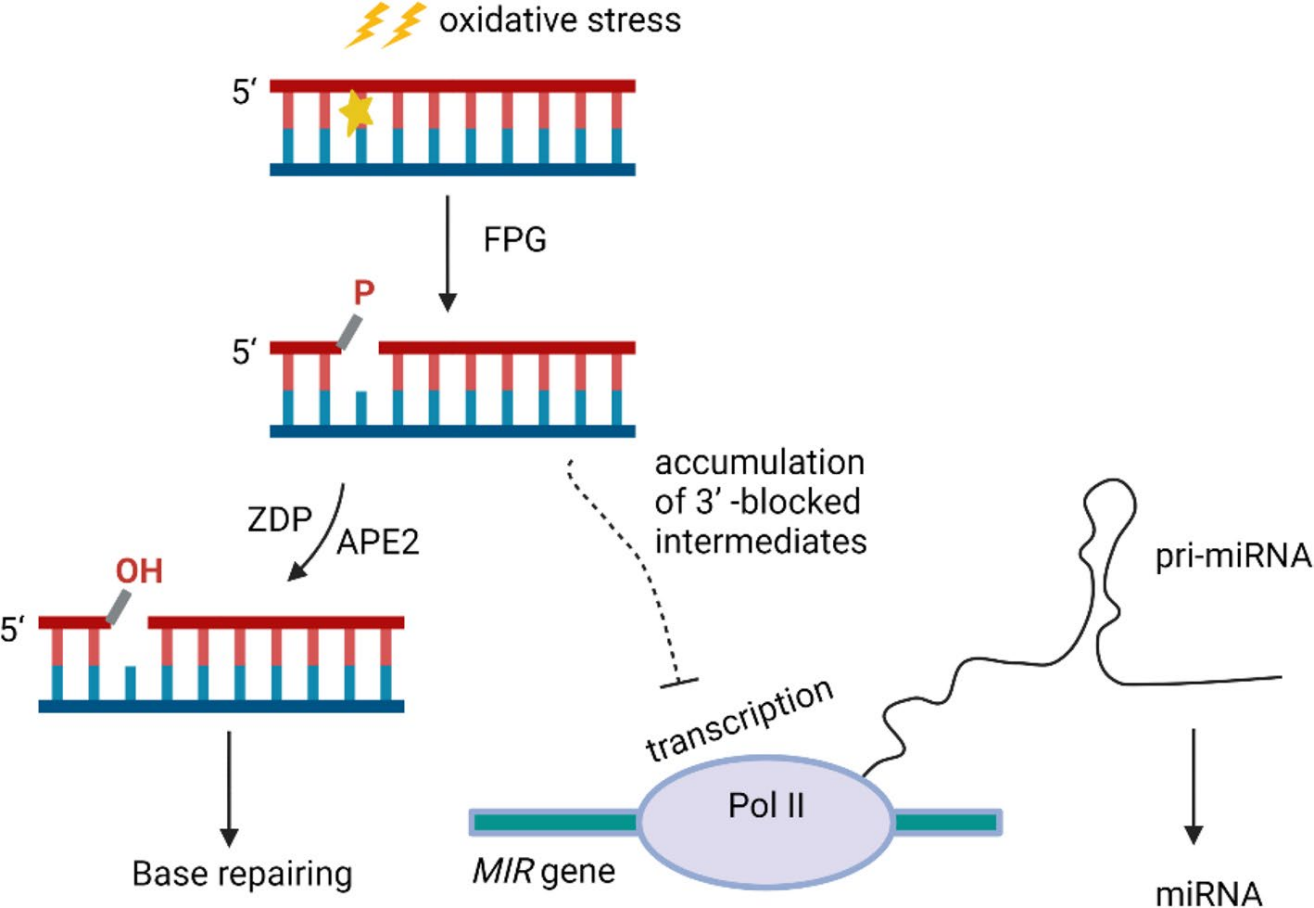
Proposed working model showing the miRNA response to DNA repair intermediates. The FPG-mediated BER pathway initiated under oxidative stress, generating blocked 3′-P end repair intermediates. In WT plants, ZDP and APE2 converted 3′-P end to 3′-OH end to allow subsequent progress in base repair. In *zdp*/*ape2* mutant, 3′-P blocked repair intermediates accumulate and lead to the inhibition of *MIR* transcription, which causes reduced accumulation of pri-miRNAs and miRNAs. The starburst indicates an oxidized base

DNA damage repair plays crucial roles in all species to maintain genome integrity. However, the molecular mechanisms of miRNA responses to DNA damage repair and the DDR in plants are unclear. In this study, we determined the reaction of miRNAs to DNA damage and found that the decrease in *MIR* transcription was accompanied by the accumulation of DNA damage repair intermediates (Fig. 5).

miRNAs react to DNA damage stress in plants. DNA double-strand breaks modulate the expression of multiple miRNAs through several mechanisms in mammals [42, 58–60]. A total of 150 miRNAs show differential expression levels in bleomycin-treated rice roots [48]. Through small RNA sequencing, we observed a genome-wide alteration of miRNAs in *zdp-1*/*ape2-2* mutant plants. Moreover, the total expression levels of downregulated miRNAs accounted for the majority of the total miRNAs that had accumulated. Abiotic and biotic stress can induce an oxidative burst that damages DNA [61, 62]. Although 4 miRNAs (miR159b, miR163, miR165a, and miR171a) detected in this study, the levels of other miRNAs, namely, miR827 and miR399 family members (miR399a, miR399c, miR399d, and miR399f), were profoundly reduced in the *zdp-1*/*ape2-2* mutant. The common features of these miRNAs include involvement in development regulation and biotic and abiotic stress responses [51–54, 63–67]. Besides, the developmental defects displayed by mutated DNA damage-related genes might be common in *Arabidopsis* [24, 68]. In addition, we noticed that the abundance of miR843, miR845a, and miR866-5p, which are involved in mediating the genome dose balance by triggering the production of epigenetically activated sRNAs to target transposable elements [69, 70], increased markedly in the *zdp-1*/*ape2-2* mutant. Therefore, our study reveals a genome-wide alteration of miRNAs in response to DNA damage.

DNA damage affects *MIR* transcription in plants. Our results reveal that the simultaneous mutation of ZDP and APE2 affects miRNA biogenesis at the transcriptional level. Studies in mammals have revealed that miR192, miR194, and miR215 are transcriptionally activated by P53 [71, 72]. Other transcription factors play major roles in the DDR, such as Myc and E2F, and induce the expression of several miRNAs in human cells [44]. The malfunction of ZDP and APE2 in BER leads to the accumulation of 3′-blocked DNA intermediates, activating the DDR [16, 26]. The DDR is ultimately involved in the transcriptional regulation of multiple genes controlling cell cycle checkpoints, DNA repair and programmed cell death [24]. DNA-dependent RNA polymerase II-mediated *MIR* transcription is regulated in a sophisticated manner in plants [33, 34]. Therefore, the transcription of *MIR* might be regulated by DNA damage and the DDR.

The accumulation of DNA repair intermediates inhibits *MIR* transcription. Unrepaired DNA lesions and intermediates initiate the DDR [24–26]. Genes involved in the DDR, including *RAD51*, *BRCA1*, *MRE11* and *WEE11*, were activated in *zdp*/*ape2* mutant under normal conditions [16]. *Arabidopsis* APE2 carries an GRF-type zinc finger domain [16], and shows high sequence similarity to human APE1 [73]. *h*APE1 functions in damaged DNA/RNA repair, and the downregulation of *h*APE1 is accompanied by alterations in miRNA expression [74, 75]. Thus, the decrease in miRNA expression in the *zdp*/*ape2* mutant in our study might have been related to the direct function of ZDP and APE2 on *MIR* transcription regulation or the indirect regulation by the DDR invoked by the accumulation of 3′-blocked DNA repair intermediates. Treatment with MMS exacerbated the decrease in miRNAs in *zdp*/*ape2* mutant, and mutation in *FPG*, which initiates DNA damage repair, rescued miRNA expression in the *zdp*/*ape2* mutant plants. These findings suggest that *MIR* transcription alterations react to the accumulation of DNA damage repair intermediates.

Animal miRNAs that respond to DNA damage can also modulate DNA damage in return [39, 76]. The interrelationship between DNA damage and miRNA biogenesis is proposed, especially with respect to the feedback loop comprising miR-17–97 and the E2F and Myc transcription factors in a cancer network [44]. A few studies in plants have also indicated that changes in accumulation of miRNAs might regulate DNA damage in feedback loop mechanism [39, 44, 48]. The DNA repair helicase *XPB2* has been predicted to be a target of miR166a-3p, and a putative suppressor of the stem-loop protein 1 *Os04g42990* has been predicted to be a target of miR167d and miR167a-5p in rice [48]*.* Our observations show that the accumulation of miR166 and miR167 is reduced in *zdp-1*/*ape2-2* mutant. These results suggest that plant miRNAs react to DNA damage and may form a feedback loop during the DDR.

## Conclusions

Our investigation suggests that DNA damage repair intermediates regulate miRNA accumulation at transcriptional level.

## Methods

### Plant materials and growth conditions

*Arabidopsis thaliana* ecotype Columbia (Col-0) served as the genetic background for all mutants. The T-DNA insertion lines *zdp-1*/*ape2-2*, *zdp-1*/*ape2-3*, *fpg-1*, *fpg-1*/*zdp-1*/*ape2-2*, and *fpg-1*/*zdp-1*/*ape2-3* have been previously reported [77]. *A. thaliana* T-DNA insertion line *ago1-27* was crossed with *zdp-1*/*ape2-2* to obtain *zdp-1*/*ape2-2*/*ago1-27* triple mutant. The transgenic *Arabidopsis thaliana* (Col-0 ecotype) line *pMIR159b::GUS* expressing GUS under the *MIR159b* promoter was a kind gift from Dr. Yijun Qi [57]. *pMIR159b::GUS* in Col-0 was crossed with *zdp-1*/*ape2-2* to generate *pMIR159b::GUS in zdp-1*/*ape2-2.* Seeds were surface-sterilized and grown on 1/2 MS medium supplemented with 1% sucrose. After 2 days incubation at 4 °C, the plates were transferred to a growth chamber under a 16 h/8 h light/dark cycle at 22 °C. For MMS sensitivity assay, seeds were grown on 1/2 MS medium supplemented with 0 or 10 ppm MMS.

### RNA extraction

Seedlings harvested from MS medium were grounded into fine powder in liquid nitrogen, and mixed with Trizol reagent (Invitrogen, 15,596,018) for RNA isolation. The solution was mixed thoroughly and added 1/5 volume of chloroform for homogenization. After incubation at RT for 5 min, the sample was centrifuged at 12,000 rpm at 4 °C for 15 min. The supernatant was mixed with 2.2-fold volume of ethanol and stored at -20 °C overnight. The mixture was then centrifuged at 12,000 rpm at 4 °C for 15 min. The pellet was washed with 75% ethanol and dissolved with RNase-free H_2_O.

### Small RNA library construction and analysis

Small RNA library was constructed as previously reported [78]. Briefly, total RNA was extracted from 2-week-old seedlings grown on 1/2 MS medium with Trizol reagent. 30 μg RNA was loaded on 15% urea-PAGE gel, and the small RNAs range from 18–30 nt were sliced from the gel. Small RNAs were recovered by soaking the smashed gel in 0.3 M NaCl overnight, followed by precipitation with ethanol. Small RNA libraries were constructed following instructions from NEBNex® Small RNA library Prep Set for Illumina® (NEB, E7300S). 4 small RNA libraries were constructed both for Col-0 and *zdp-1*/*ape2-2* samples. The small RNA libraries were single-end sequenced on an Illumina HiSeq2500 platform.

Small RNAs sequences were processed with Cutadapt v3.4 [79] to remove sequencing adaptors and low-quality bases. Reads with length between 18 to 50 nt were retained for further analyses. Clean reads were mapped to the *Arabidopsis* genome (TAIR10 version) with SPORT v1.1.1 [80] and were annotated into non-coding RNA categories, including miRNA, tRNA, rRNA, siRNA, etc. To perform differential expression analysis of miRNA, clean reads were mapped to the *Arabidopsis* genome (TAIR10 version) with bowtie v1.3.0 [81] allowing no mismatches. Uniquely mapped reads were used to calculate miRNA counts with featureCounts v2.0.1 [82]. miRNA abundance was normolized to reads per million with the sum of 18-30 nt reads. Fold-change of RPM between Col-0 and *zdp-1*/*ape2-2* was calculated for each miRNA. Fold-change ≥ 1.5 or ≤ -1.5 were used as threshold for differential expressed miRNAs. Correlation was calculated using Euclidean’s distance matrix using PtR program in Trinity package [83].

### RT‒qPCR

Expression levels of pri-miRNAs, mRNAs, and miRNAs were examined by quantitative real-time PCR. Total RNA was extracted using Trizol reagent from 2-week-old seedlings grown on 1/2 MS medium. For mRNA reverse transcription, cDNA was synthesized in 20 μl reaction volumes using 1 μg DNase-I (NEB, M0303) treated total RNA and reversely transcribed with the *Evo M-MLV* Mix Kit (AIKERUI, AG11728). For miRNA reverse transcription, 1 ug of total RNA was digested with DNase I and poly (A) was added to the 3′ end by* E. coli* poly (A)  Polymerase (NEB, M0276). The first-strand cDNAs were transcribed by M-MuLV reverse transcriptase (NEB, M0253). RT‒qPCR was performed in 10 μl volumes containing 2 μl of 20-fold diluted cDNA, 5 μl of SYBR Green mix (Vazyme, Q311), and 0.2 μM of each primer. The analysis was performed in One-way PCR detection system (Invitrogen) using the following cycling conditions: initial denaturation at 95 °C for 30 s, followed by 40 cycles of 95 °C for 5 s and 60 °C for 30 s. All data was normalized to EF1α. Primers used in RT‒qPCR are listed in Supplementary Table S4. The relative fold change in the expression levels were calculated using the 2^−∆∆Ct^ method. All reactions were carried out in 3 biological replicates.

### Northern blot

Northern blot was performed as described [35, 84]. Total RNA was separated on 14% denaturing urea-polyacrylamine gels and run with 0.5 × TBE at 150 V. The gel was transferred to Hybond membrane NX (GE healthcare, RPN303T) at 14 V overnight. Chemical crosslink buffer was prepared as follows: 0.373 g N-(3-Dimethylaminopropyl)-N′-ethylcarbodiimide hydrochloride (Sigma-Aldrich, E7550), 3 drops of 1 M HCl, 121 µl Methylimidazole (Sigma-Aldrich, M50834), and 12 mL RNase-free H_2_O. After chemical crosslink at 60 °C for 2 h and UV crosslink at 85 °C for 2 h, the membrane was pre-incubated with PerfectHyb™ Plus Hybridization Buffer liquid (Sigma-Aldrich, H7033) for 30 min, then hybridized overnight at 37 °C with γ- ^32^P ATP (China isotope & radiation corporation) labelled DNA probes by T4-polynucleotide kinase (NEB, M0201S) for 4 h. After that, the membrane was washed with buffer contains 2 × SSC and 0.025% SDS. Auto-radiography of the membrane was performed using a Typhoon Scanner. Sequences of probes are listed in Supplementary Table S4.

### Nuclear run on assay

Nuclear run-on assay was performed as described [85, 86]. Briefly, 0.5 g 2-week-old seedlings were harvested and grounded into fine powder in liquid nitrogen and mixed with pre-cooled nuclease-free Lysis buffer (20 mM Tris–HCl, pH 7.5, 20 mM KCl, 2 mM EDTA, 2.5 mM MgCl_2_, 25% glycerol, 250 mM Sucrose, and 5 mM DTT). The homogenate was filtered through a double layer of miracloth (Merck, 475,855). The flow-through was spun at 2000 g for 10 min at 4 °C. The pellet was washed 2–3 times with NRBT buffer (20 mM Tris–HCl, pH 7.5, 25% glycerol, 2.5 mM MgCl_2_, 0.2% Triton X-100, and 4 mM DTT) and resuspended in 50 μl nuclei storage buffer (50 mM Tris–HCl, pH 7.5, 1 mM DTT, 20% Glycerol, 5 mM MgCl_2,_ and 0.44 M Sucrose). The run-on assay was performed in 1 × transcription assay buffer (50 mM Tris–HCl, pH 7.5, 5 mM MgCl_2,_ 150 mM KCl, 0.1% sarkosyl, 2 U/ml RNase inhibitor, 10 mM DTT, 10 mM rATP, 10 mM rCTP, 10 mM rGTP, and 10 mM BrUTP (Sigma-Aldrich, B7166)] at 30 °C for 30 min. The reaction was stopped by adding 600 μl Trizol reagent, and RNAs were extracted and treated with DNase I to remove genomic DNA. The purified RNAs were diluted in 500 μl incubation buffer (0.25 × SSPE, 0.05% Tween-20, 37.5 mM NaCl, and 1 mM EDTA) and incubate with 2 μg anti-BrdU antibody (Sigma-Aldrich, B8434) at 4 °C for 2 h and then subjected to immunoprecipitation for 1 h with Dynabeads protein G (Invitrogen, 1003D) pre-coated with yeast tRNA (Invitrogen, AM7119). The precipitated beads were washed with low salt buffer (0.2 × SSPE, 1 mM EDTA, 0.05% Tween-20) twice, followed by washes with high salt buffer (0.5 × SPPE, 1 mM EDTA, 0.05% Tween-20, 150 mM NaCl) twice. The precipitated RNAs were extracted by Trizol reagent and used for cDNA synthesis and RT‒qPCR analysis.

### GUS staining

GUS staining was performed with 2-week-old plants. Seedlings were immersed in GUS staining solution and incubated at 37 °C overnight. After staining, rinse the seedlings in 75% ethanol until the clear of chlorophyll. Pictures were taken under stereo microscope.

## Supplementary Information

**Additional file 1:** **Supplementary Fig. S1.** Summary of sRNA sequencing data and heatmap of the Pearson correlation between the expression level of miRNAs in Col-0 and *zdp-1*/*ape2-2* mutant. 

**Additional file 2:** **Supplementary Fig S2.** Full-length blots in Northern Blot assays of miRNAs. 

**Additional file 3:** **Supplementary Fig. S3.** Genetic interactions between ZDP and APE2 and AGO1. 

**Additional file 4:** **Supplementary Fig. S4.** Snapshot of methylation levels on *MIR*s in Col-0 and *zdp-1*/*ape2-2* mutant. 

**Additional file 5:** **Supplementary Table S1.** Summary of sRNA-seq library in Col-0 and *zdp-1*/*ape2-2*. 

**Additional file 6:** **Supplementary Table S2.** 18-30 nt sRNAs length distribution in Col-0 and *zdp-1*/*ape2-2* sRNA libraries.

**Additional file 7:** **Supplementary Table S3.** miRNAs abundance in Col-0 and *zdp-1*/*ape2-2 *in sRNA libraries. 

**Additional file 8**: **Supplementary Table S4.** List of primers used in this study.

**Additional file 9:** **Supplementary Table S5.** Statistical analysis in Fig. 1. 

**Additional file 10:** **Supplementary Table S6.** Statistical analysis in Fig. 2. 

**Additional file 11:** **Supplementary Table S7.** Statistical analysis in Fig. 3. 

**Additional file 12:** **Supplementary Table S8.** Statistical analysis in Fig. 4.

## Data Availability

The raw sequences have been deposited in the National Center for Biotechnology Information SRA (accession no. SRR19546282-SRR19546289, SRR22439143-SRR22439144) or BioProject (accession no. PRJNA846179 with reviewers’ link https://dataview.ncbi.nlm.nih.gov/object/PRJNA846179?reviewer=15s0rdqavjluq5ec7e55djmhor)). All study data are available in the main text or supplementary materials.

## Abbreviations

miRNAs: MicroRNAs
ZDP: Zinc finger DNA 3′-phosphoesterase
APE2: AP endonuclease 2
BER: Base excision repair
*MIR*: MiRNA encoding gene
MMS: Methyl methanesulfonate
FPG: Formamidopyrimidine DNA Glycosylase
ROS: Reactive oxygen species
AP: Apurinic/apyrimidinic
3′-PUA: 3′-Phosphor-α, β-unsaturated aldehyde
3′-P: 3′-Phosphate
5′-OH: 5′-Hydroxyl
ROS1: Repressor of Silencing 1
ATM: Ataxia telangiectasia mutated
nt: Nucleotide
pri-miRNAs: Primary miRNAs
DDR: DNA damage response
AGO1: ARGONAUTE 1
sRNAs: Small RNAs
