# Supplementary material for: Accumulation of DNA damage alters microRNA gene transcription in Arabidopsis thaliana
Source: BMC Plant Biol. 2022 Dec 12;22:576. doi: 10.1186/s12870-022-03951-9 (PMC9743578; doi:10.1186/s12870-022-03951-9)
Supplement: Supplementary file 1 — Additional file 1: Supplementary Fig. S1. Summary of sRNA sequencing data and heatmap of the Pearson correlation between the expression level of miRNAs in Col-0 and zdp-1/ape2-2 mutant. [file 12870_2022_3951_MOESM1_ESM.docx]

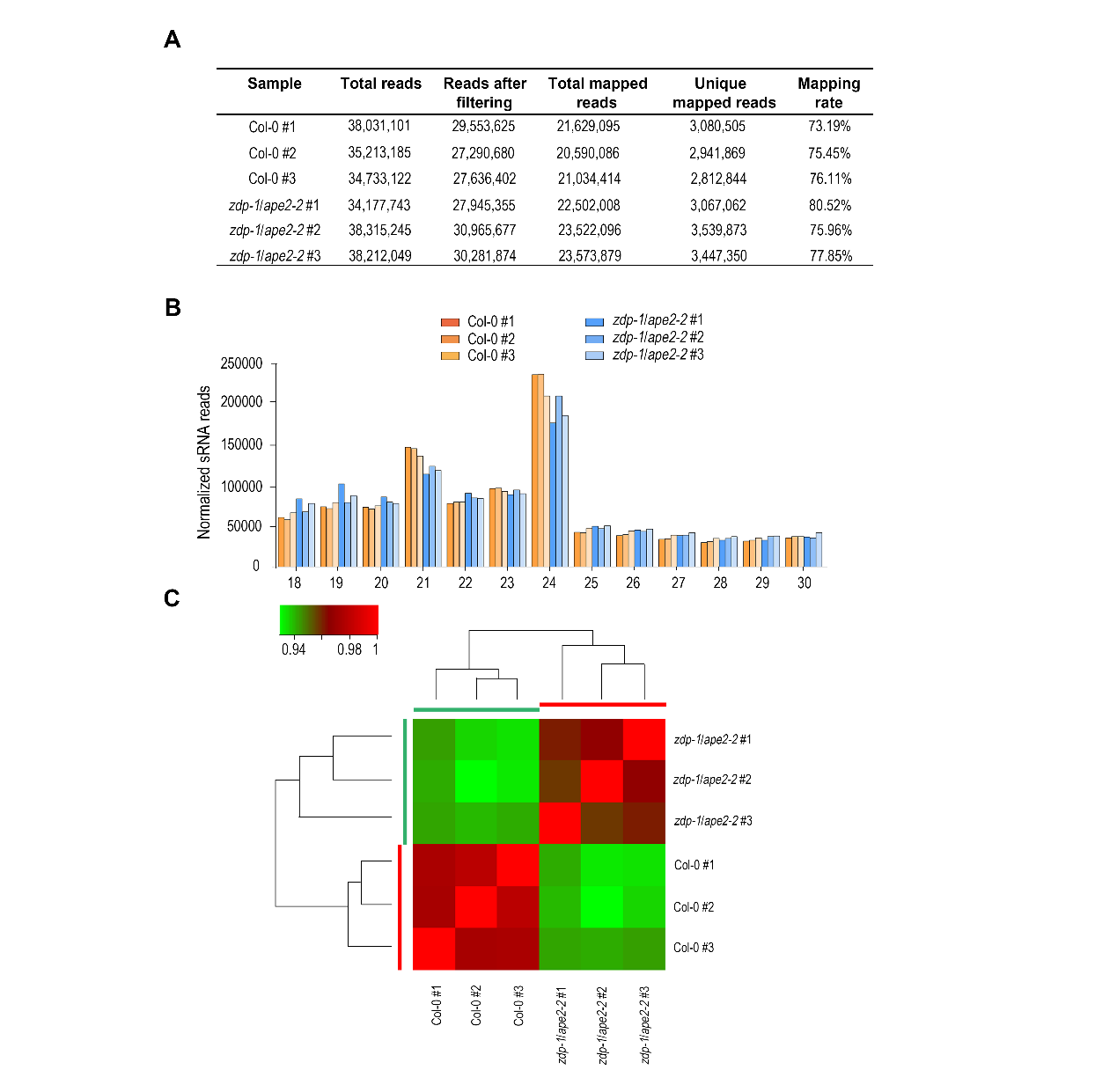


**Supplementary Dataset File 1, Supplementary Fig. S1.** Summary of sRNA sequencing data and heatmap of the Pearson correlation between the expression level of miRNAs in Col-0 and *zdp-1*/*ape2-2* mutant. Related to Fig. 1. **A:** Summary of sRNA sequencing data in Col-0 and *zdp-1*/*ape2-2* mutant plants. #1, #2, and #3 mean three biological repeats. **B:** The length distribution of small RNAs in Col-0 and *zdp-1*/*ape2-2*. Each sample has three biological repeats. **C:** Heatmap of the Pearson correlation between the expression levels of miRNAs in Col-0 and *zdp-1*/*ape2-2* mutant plants. Red color represents higher correlation while green color represents lower correlation.
