# Supplementary material for: Accumulation of DNA damage alters microRNA gene transcription in Arabidopsis thaliana
Source: BMC Plant Biol. 2022 Dec 12;22:576. doi: 10.1186/s12870-022-03951-9 (PMC9743578; doi:10.1186/s12870-022-03951-9)
Supplement: Supplementary file 2 — Additional file 2: Supplementary Fig S2. Full-length blots in Northern Blot assays of miRNAs. [file 12870_2022_3951_MOESM2_ESM.docx]

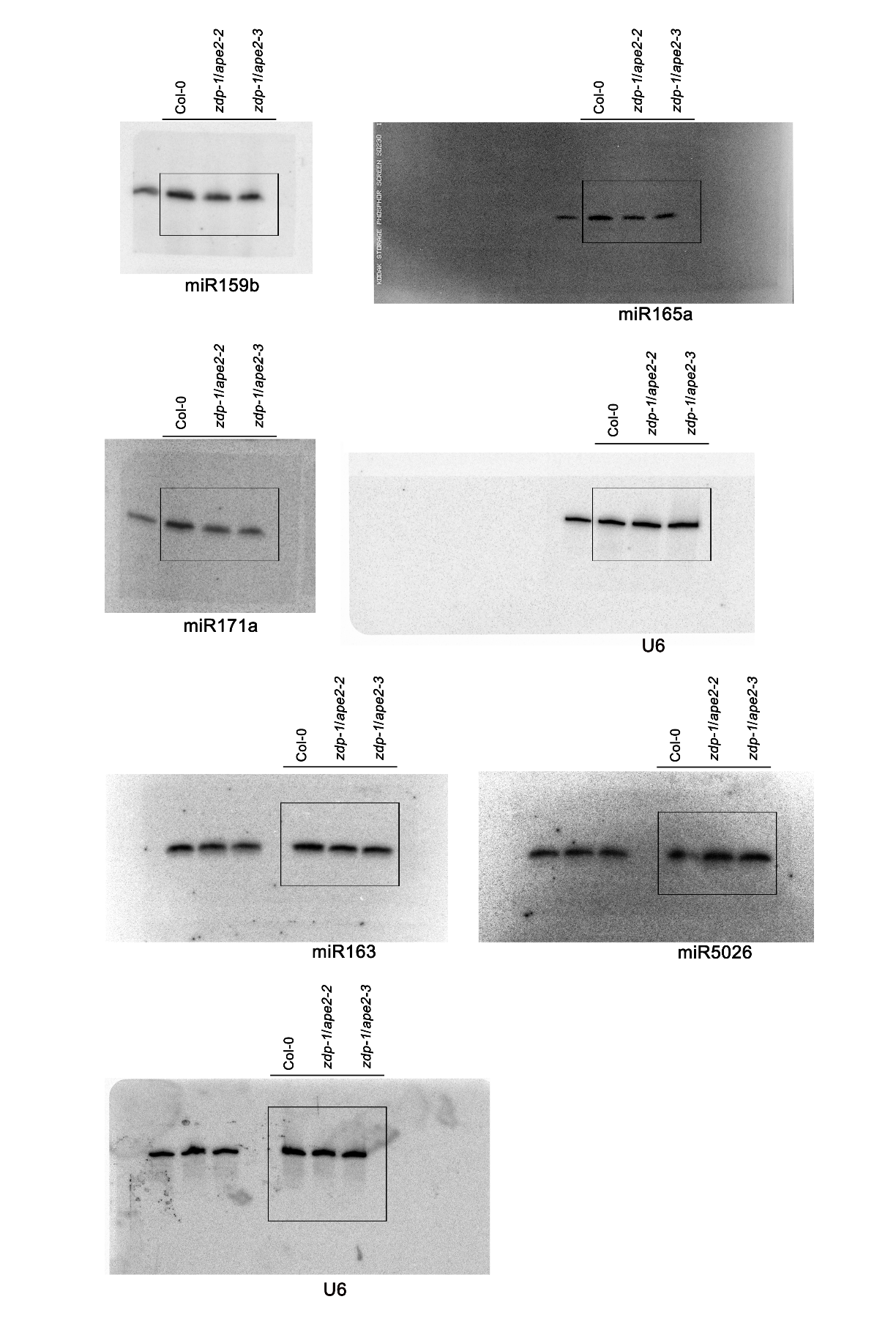


**Supplementary Dataset File 2, Supplementary Fig. S2.** Full-length blots in Northern Blot assays of miRNAs.
