# Supplementary material for: Accumulation of DNA damage alters microRNA gene transcription in Arabidopsis thaliana
Source: BMC Plant Biol. 2022 Dec 12;22:576. doi: 10.1186/s12870-022-03951-9 (PMC9743578; doi:10.1186/s12870-022-03951-9)
Supplement: Supplementary file 3 — Additional file 3: Supplementary Fig. S3. Genetic interactions between ZDP and APE2 and AGO1. [file 12870_2022_3951_MOESM3_ESM.docx]

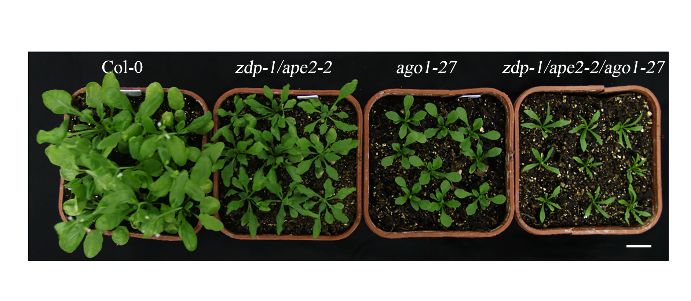


**Supplementary Dataset File 3, Supplementary Fig. S3.** Genetic interactions between ZDP and APE2 and AGO1. The triple homozygous mutants grown on MS/2 medium or soil show additive developmental defects compared with respective single or double mutants. Developmental phenotype of Col-0, *zdp-1*/*ape2-2*, *ago1-27*, *zdp-1*/*ape2-2*/*ago1-27*. 2-week-old seedlings were grown on MS/2 medium. Scale bars represent 1cm.
