# Supplementary material for: Accumulation of DNA damage alters microRNA gene transcription in Arabidopsis thaliana
Source: BMC Plant Biol. 2022 Dec 12;22:576. doi: 10.1186/s12870-022-03951-9 (PMC9743578; doi:10.1186/s12870-022-03951-9)
Supplement: Supplementary file 4 — Additional file 4: Supplementary Fig. S4. Snapshot of methylation levels on MIRs in Col-0 and zdp-1/ape2-2 mutant. [file 12870_2022_3951_MOESM4_ESM.docx]

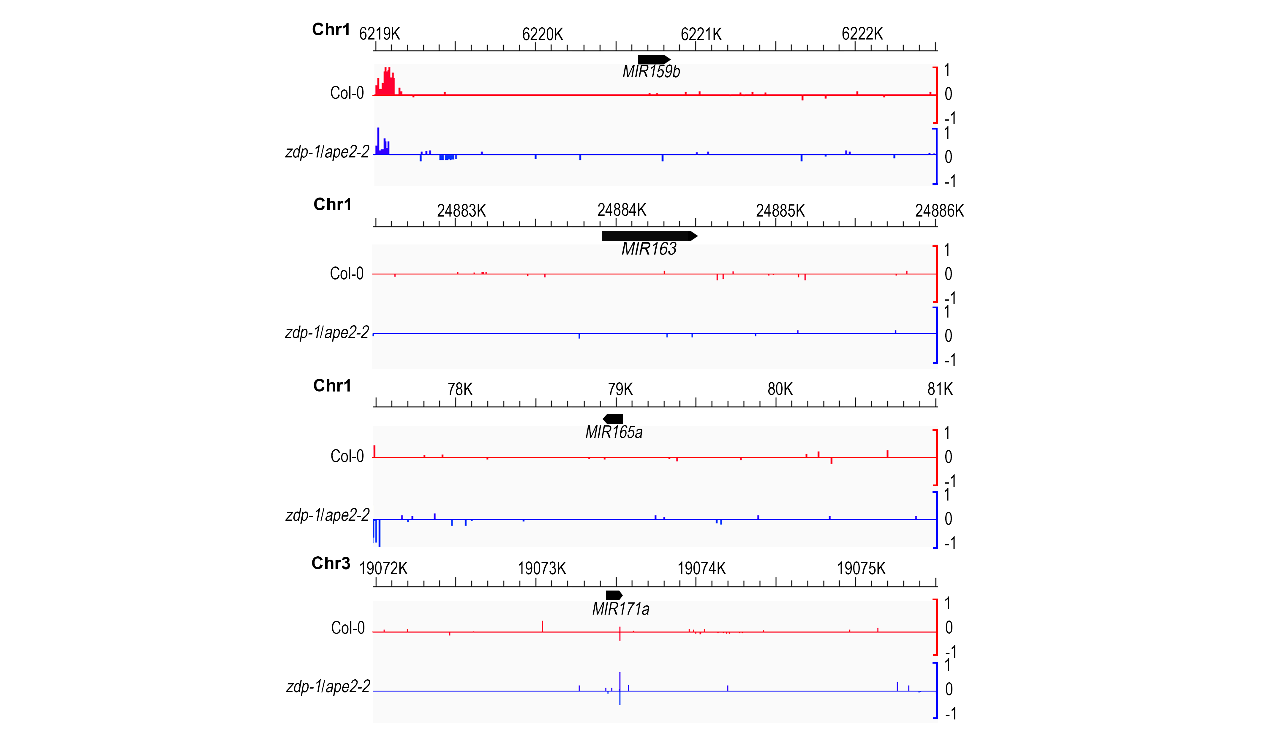


**Supplementary Dataset File 4, Supplementary Fig. S4.** Snapshot of methylation levels on *MIR*s in Col-0 and *zdp-1*/*ape2-2* mutant. Related to Fig. 3. Data from WGBS (Whole Genome Bisulfite Sequencing, SRA accession no. SRP119987 or BioProject accession no. PRJNA412269)
